# An interactive motion perception tool for kindergarteners (and vision scientists)

**DOI:** 10.1177/20416695231159182

**Published:** 2023-03-30

**Authors:** Aravind Battaje, Oliver Brock, Martin Rolfs

**Affiliations:** Robotics and Biology Laboratory, 26524 Technische Universität Berlin, Germany; Science of Intelligence, Research Cluster of Excellence, Berlin, Germany; Robotics and Biology Laboratory, 26524 Technische Universität Berlin, Germany; Science of Intelligence, Research Cluster of Excellence, Berlin, Germany; Department of Psychology, 9373 Humboldt-Universität zu Berlin, Germany; Science of Intelligence, Research Cluster of Excellence, Berlin, Germany

**Keywords:** spatiotemporal filtering, motion energy, interactive, visual illusions, science education, eye movement

## Abstract

We implement Adelson and Bergen's spatiotemporal energy model with extension to three-dimensional (x–y–t) in an interactive tool. It helps gain an easy understanding of early (first-order) visual motion perception. We demonstrate its usefulness in explaining an assortment of phenomena, including some that are typically not associated with the spatiotemporal energy model.

We present a tool^1^ that reveals key characteristics of early visual motion perception through a simple inspection. The setup involves pointing an off-the-shelf camera at visual images or videos such as in Figure 1. This tool implements the spatiotemporal energy model^2^ (Adelson & Bergen, 1985; Watson & Ahumada, 1985)—the standard model of first-order motion perception (Nishida et al., 2018)—with extension to three-dimensional (3D, x–y–t) in real-time, and visualizes perceived motion direction by mapping it on to a color wheel (Figure 2). This makes gaining insights about early visual motion perception an easy and interactive experience.^3^

**Figure 1.**
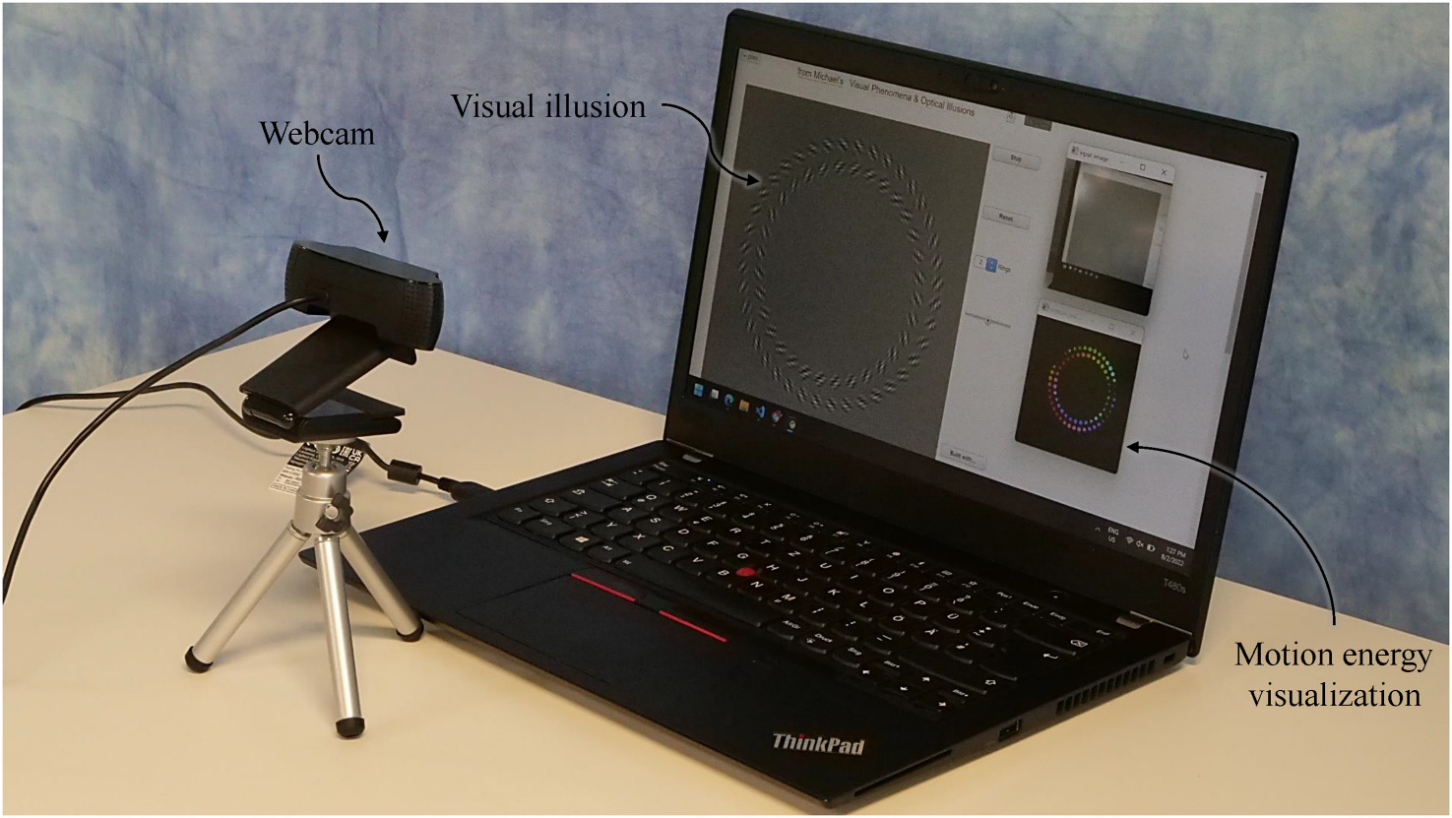
A laptop and a webcam explain motion visual illusions. Here, the illusory rotation of the rings in Pinna–Brelstaff illusion (Pinna & Brelstaff, 2000) is immediately revealed through the colors encoding motion energy (Adelson & Bergen, 1985). Refer to Figure 2 for more details.

**Figure 2.**
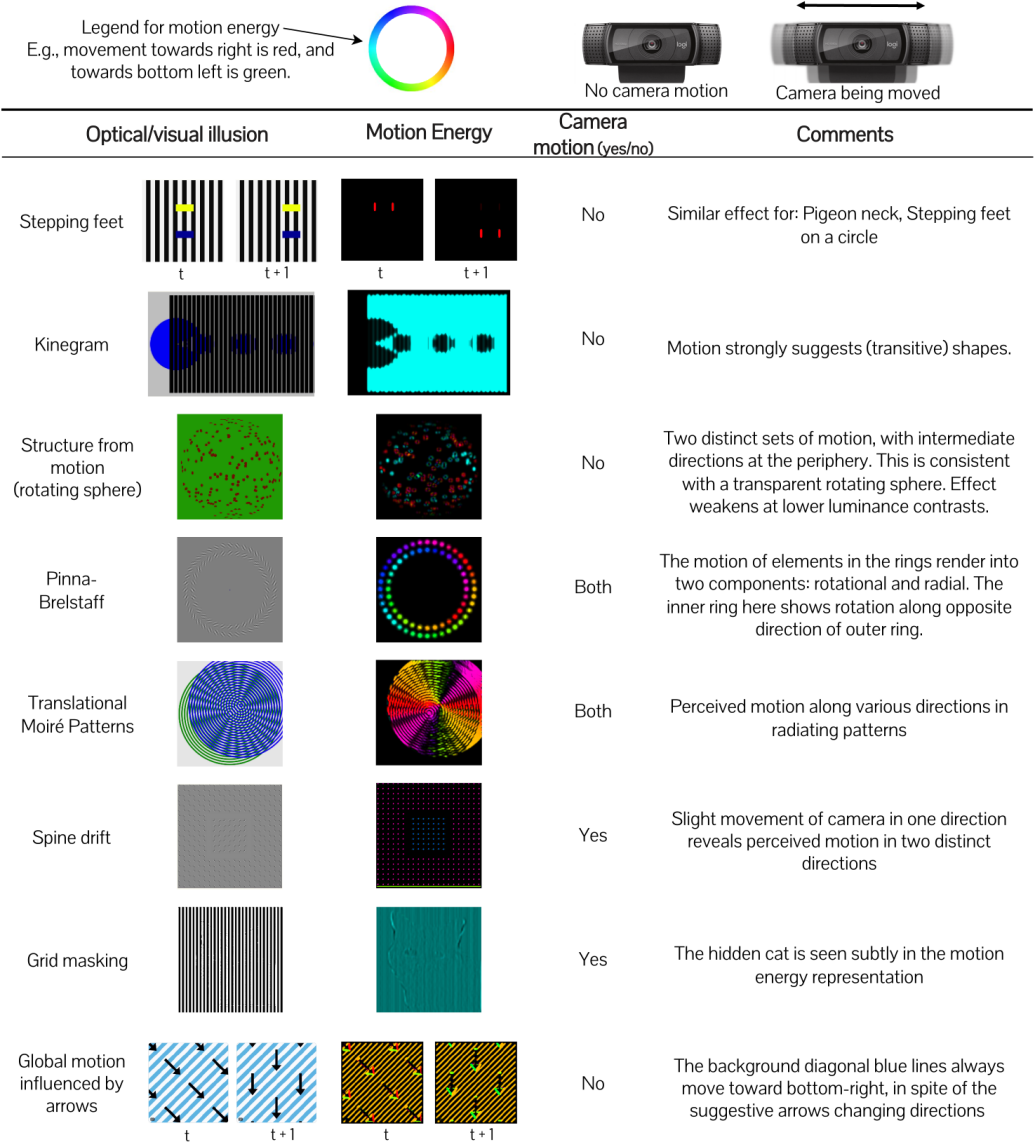
The motion perception tool explains an assortment of visual illusions: stepping feet (Anstis, 2001; Bach, 2004; Kitaoka & Anstis, 2021), Kinegram (Bach, 2014), structure from motion (Bach, 2002; Rogers & Graham, 1979), Pinna–Brelstaff (Bach, 2003; Pinna & Brelstaff, 2000), Translational Moirè Patterns (Bach, 2013; Spillmann, 1993), Spine drift (Bach, 2011; Kitaoka, 2010), grid masking (Bach, 2019), and global motion influenced by arrows (@jagarikin, 2022).

In fact, by simply playing around with the tool, we discovered that spatiotemporal energy models (Adelson & Bergen, 1985) directly explain many more phenomena than previously understood^4^ . We applied our tool to illusions available on YouTube and Twitter, as well as curated lists (Bach, 1997; Shapiro & Todorovic, 2017). For some of them, we also moved the camera to mimic head/eye movements. In Figure 2, we show outputs on an assorted list of phenomena we have found in this process.

This is in contrast to traditional methods of testing a model on visual stimuli. Instead of saving image sequences to disk, processing them offline, and generating visualizations of energy for different motion directions over time (as a post-processing step), our tool allows doing all this live.

Take, for example, the **stepping feet illusion** (Anstis, 2001). In this illusion (Bach, 2004: shows a demo), the yellow and blue “feet” are set against a grating of black-and-white lines. The “feet” are vertically aligned, and move smoothly and at the same speed. But they produce an illusion of stepping alternatively as if the yellow foot pauses when the blue foot moves and vice versa. When we point a webcam at this illusion and run our motion perception tool, it directly and immediately reflects our perception (row 1 of Figure 2). The “feet” appear to move in distinct steps, at consecutive times^5^ t and t+1. You may also reduce the contrast of grating (Bach, 2004: un-tick the “Hi contrast” button), and notice that it weakens the strength of the illusion. With this, it is easy to understand the role of contrast in motion perception (Anstis, 2004).

Another example is the **Pinna–Brelstaff illusion** (Pinna & Brelstaff, 2000). First, let us look at the animated online version of this illusion (Bach, 2003) that simulates the head moving toward/away (looming) with respect to the static pattern. Here, even though the rings are simply expanding or contracting, it elicits additional illusory rotations for each ring. We can verify this same percept with our tool (row 4 of Figure 2).

To understand the visualization, the notion of a “phase” for rings is helpful. On an expanding ring without rotation, every point will move away from its center in the same direction of the *radial* line^6^ . On the other hand, a rotating ring produces motion along the *tangent* at every point. Thus, the visual motion “phase” of a rotating ring relates to an expanding ring in the following way: taking the expanding ring as a reference ( $0^{\circ }$), a clockwise rotating ring has a phase of $+90^{\circ }$. In other words, a clockwise rotating ring is $+90^{\circ }$ rotation of an expanding ring. Likewise, a counter-clockwise rotating ring has a phase of $-90^{\circ }$. Any intermediate phase indicates simultaneous rotation and expansion as the resultant phase is a vector addition of movement due to expansion and rotation.

For the animated version of the Pinna–Brelstaff illusion (Bach, 2003), the output of our motion perception tool is a combination of radial and tangential motion. When the rings are expanding, the inner ring has a phase of $+45^{\circ }$ (illusory clockwise rotation), and the outer ring has a phase of $-45^{\circ }$ (illusory counter-clockwise rotation). You may further change the angle of Gabor elements, using the slider on the right, and immediately notice its effect on the strength and direction of illusory rotations.

Static patterns of the Pinna–Brelstaff illusion (Pinna & Brelstaff, 2000) also elicit an illusory percept for translation and rotations of the head. This too can be reproduced with our tool by simply moving the camera, roughly recreating the required head motion. The ability to interact with the visual illusion by moving the camera is powerful as it allows us to understand action–perception coupling (Rolfs & Schweitzer, 2022) for the case of self-movement and visual motion perception.

The **spine drift illusion** (Bach, 2011; Kitaoka, 2010: shows a demo) demonstrates such a relationship between eye movements and motion perception (Menshikova & Krivykh, 2016). When we view this illusion, the central square appears to float with respect to the background. We can reproduce this by slightly moving the camera, which simulates fixational eye movements (Rolfs, 2009). This produces perceived motion along two distinct directions (row 6 of Figure 2), indicating that the center square excites motion receptors strongly in a direction different from that of the periphery.

In this paper, we explored a limited range of phenomena—some with imprecise head/eye movements. However, our tool easily extends to study other scenarios. You may use it to study motion perception during smooth pursuit (Battaje & Brock, 2022; Morvan & Wexler, 2009; Terao et al., 2015), or with slow erratic drift and miniature saccades (Rolfs, 2009), which are known to contribute to many motion illusions (Beer et al., 2008; Menshikova & Krivykh, 2016; Murakami, 2006; Troncoso et al., 2008). Or alternatively, to study illusions based on eye blinks (Faubert & Herbert, 1999; Otero-Millan et al., 2012).

The key to the usefulness of our tool is its ability to run in real-time. For this, we use PyTorch (Paszke et al., 2019), a library targeted toward deep learning. It makes low-level accelerated computing routines^7^ accessible through a high-level programming language. With a few lines of code, it is easy to apply linear filtering (convolutions) on a sequence of images—a spatiotemporal volume—in real-time. For example, our tool is a template for a component of an active vision robotic application (Battaje & Brock, 2022) that uses fixation and resultant motion cues for 3D perception.

Similarly, we believe the interactive real-time nature of this tool could be extended to other domains. From color perception to the perception of causality and animacy, when there are computational models that may be expressed as linear filters (for which computation is fast), it would be easy to implement tools similar to the one described here, and immediately “see” the results of a given model.

In conclusion, we present an interactive tool that helps explain early visual motion perception. The setup is simple: a laptop and an external webcam. Using this tool, we can easily explain old, as well as new, visual phenomena. This also works for phenomena that involve physical eye movements. The code is openly available and uses accelerated computing libraries that make it easy to adapt to other, more complex visual perception models. With this, the process of learning and discovery becomes as simple as playing with toys. We hope the vision science community can take advantage of such a method of interactive discovery.

## Supplemental Material

sj-pdf-1-ipe-10.1177_20416695231159182 - Supplemental material for An interactive motion perception tool for kindergarteners (and vision scientists) Click here for additional data file. Supplemental material, sj-pdf-1-ipe-10.1177_20416695231159182 for An interactive motion perception tool for kindergarteners (and vision scientists) by Aravind Battaje, Oliver Brock and Martin Rolfs in i-Perception

## Notes

1. The code is available at https://github.com/aravindbattaje/motion_perception_tool.

2. We use temporal and spatial filter parameters similar to (Challinor & Mather, 2010; Mather & Challinor, 2009). For more implementation details, including an extension of the model to three-dimensional (3D), refer to the “Implementation Details” section of Supplementary Paper 01.

3. Refer to Video 01 and Video 02 in online Supplemental Material, to see this in action.

4. We validate our model with stimuli containing continuous motion, sampled motion, reverse-phi illusory motion, and flute square wave illusion, as done in Adelson and Bergen (1985). Details of validation can be found in the “Model Validation” section of Supplemental Paper 01.

5. Units of time are representative, scaled to the speed of the “feet”.

6. The “legend for motion energy” on the top of Figure 2 corresponds to an expanding ring.

7. Our tool uses Central Processing Unit accelerations by default. But we also provide an option to switch to Graphics Processing Unit accelerations, to demonstrate its applicability to high image resolutions and more complex models.
