# Supplementary material for: An interactive motion perception tool for kindergarteners (and vision scientists)
Source: Iperception. 2023 Mar 30;14(2):20416695231159182. doi: 10.1177/20416695231159182 (PMC10064475; doi:10.1177/20416695231159182)
Supplement: sj-pdf-1-ipe-10.1177_20416695231159182 - Supplemental material for An interactive motion perception tool for kindergarteners (and vision scientists) [file sj-pdf-1-ipe-10.1177_20416695231159182.pdf]

Journal Title
XX(X):1–4

©The Author(s) 2022
Reprints and permission:
sagepub.co.uk/journalsPermissions.nav
DOI: 10.1177/ToBeAssigned
www.sagepub.com/
SAGE

Supplementary Material for *An Interactive Motion Perception Tool for Kindergarteners (and Vision Scientists)*

Aravind Battaje [a, c], Oliver Brock [a, c] and Martin Rolfs [b, c]

Implementation Details

In our interactive motion perception tool, we implement the spatiotemporal energy model (Adelson & Bergen, 1985) in 3D (x-y-t), building upon previous computational implementations (Mather & Challinor, 2009; Challinor & Mather, 2010). In the following, we first describe the model as implemented previously, and then detail our changes and extensions.

Existing Computational Model

Motion energy is calculated by filtering a spatiotemporal volume with a quadrature pair of spatiotemporal filters, and adding the squared responses of each (Adelson & Bergen, 1985). The filters are separable into spatial and temporal profiles by construction, and although the form for these separated filters were provided in the original work, the exact parameters were not provided. However, recent works (Mather & Challinor, 2009; Challinor & Mather, 2010) found suitable parameters.

The temporal filter has the following form:

$$R(t) = (kt)^n \cdot \exp(-kt) \cdot [1/n! - \beta(kt)^2/(n+2)!] \quad (1)$$

[a] Robotics and Biology Laboratory, Technische Universität Berlin
[b] Department of Psychology, Humboldt-Universität zu Berlin
[c] Science of Intelligence, Research Cluster of Excellence, Berlin

Corresponding author:
Aravind Battaje, Robotics and Biology Laboratory, Technische Universität Berlin
Email: battaje@tu-berlin.de

where t represents the time, k and n parameterize the center temporal frequency of the filter, and β controls the weighting of negative phase of the temporal impulse response function relative to its positive phase. Challinor and Mather (2010) varied k for different conditions, while keeping the other parameters fixed at $n = 9$ for slow temporal filter, $n = 6$ for fast temporal filter, and $\beta = 0.9$.

The spatial filter is composed of even (E) and odd (O) Gabor functions, forming a quadrature pair:

$$E(x) = \cos(2\pi f x) \cdot \exp(x/\sigma)^2 \tag{2}$$

$$O(x) = \sin(2\pi f x) \cdot \exp(x/\sigma)^2 \tag{3}$$

where f is the spatial frequency and σ characterizes the width of Gaussian window for Gabor functions. Challinor and Mather (2010) used $f = 1$ cpd and $\sigma = 0.5°$.

Our Implementation

We implemented the temporal filter according to Eqn. 1, with different parameter values: $k = 125$, $n = 6$ for slow temporal filter, $n = 3$ for fast temporal filter, and $\beta = 0.99$. Moreover, we extended the spatial filter to 2D (x-y) using a 2D Gabor kernel:

$$G(\boldsymbol{x}) = \mathcal{N}(\boldsymbol{0}, \boldsymbol{\Sigma}) \cdot \exp\left(i \cdot 2\pi f \cdot [\cos\theta, \sin\theta]^T \boldsymbol{x}\right) \tag{4}$$

which is a complex sinusoidal plane wave windowed through a zero-mean Gaussian function. The real and imaginary part of this expression are the 2D equivalents of Eqns. 2 and 3 respectively. Here $\boldsymbol{x}$ is a 2D vector representing spatial location $[x, y]^T$, $\boldsymbol{\Sigma}$ is the 2D covariance matrix representing the size of the window, f is the spatial frequency of the planar wave, and θ is its orientation. We use $\boldsymbol{\Sigma} = \mathrm{diag}([1.87\ \mathrm{px}, 1.87\ \mathrm{px}])$, $f = 0.3\ \mathrm{px}^{-1}$, and 4 different orientations $\theta = 0°, 45°, 90°, 135°$, which cover motion along 8 different directions. We do not express spatial quantities in relative visual angle, but this can be derived for specific applications with a fixed camera and display setup.

Using the above temporal and spatial filters, we obtain spatiotemporal energy for each orientation and simply add them up to obtain the total energy. This is possible because each orientation measures energy in 2 directions. For example, with spatial filter of $\theta = 0°$, the spatiotemporal energy is measured in left-right directions, and likewise with $\theta = 90°$, the same is measured in up-down directions. For each orientation θ, we then place the response on an axis on the X-Y plane rotated to the specified orientation. Finally, we perform vector addition of the responses from all orientations. Please note, we perform these operations mainly for illustrative purposes. We do not attempt to answer the motion integration question, such as in the velocity-space model (Adelson & Movshon, 1986). Nevertheless, our results do not depend crucially on the form of computation.~~Thus, spatiotemporal energy can be straightforwardly accumulated across all orientations.~~

To visualize spatiotemporal energy, we convert the vector sum of responses into polar coordinates and map that onto a color wheel (placed in Fig. 2 of the main paper). We map the direction to hue, and magnitude to saturation. With this scheme, each hue corresponds to a different direction and the strength of response corresponds to brightness.

The filtering operations are also completely separable, allowing for efficient computation. That is, the spatial Gabor kernel is separable into two 1D (complex) filters, and the temporal filter is separated by construction. Thus, the costliest part of the computation can be reduced to 1D convolutions. This permits real-time computation of spatiotemporal energy in even lightly-powered modern computers.

The parameters we present above work well with a fixed video resolution of 240 x 240 px at 30 and 60 fps (frames-per-second). For different video resolutions, the Gabor kernel frequencies need to be scaled accordingly.

Model Validation

To validate our implementation, we subject it to four types of real and apparent motion, as originally done in Adelson and Bergen (1985, Sec. 9). We find that our implementation faithfully reproduces motion energy for all four scenarios. We recreate each scenario by simulating 1D motion (effectively x-t). However, note that the same results can be extrapolated to any implemented orientation for 2D motions (x-y-t). In the following, we briefly describe and provide results for each scenario.

Continuous Motion A basic test for a motion-detecting system is to respond to ordinary continuous motion. In Sup. Fig. 1 (Column 1), we show that for slow movements, our model detects motion in the appropriate directions without artefacts.

Sampled Motion When the motion gets faster, the temporal sampling rate effectively drops. This results in sampling artefacts as shown in Sup. Fig. 1 (Column 2). The artefacts manifest as small amount of motion energy in the opposite direction superimposed with detected motion in the appropriate direction.

Reverse Phi Just as with continuous motion, a sufficiently slow moving random pattern should elicit motion energy in the correct direction, as shown in Sup. Fig. 2 (Column 1). However, when the polarity (or contrast) of the random pattern is reversed every frame, it results in illusory movement in the opposite (left) direction (Anstis, 1970; Anstis & Rogers, 1986) as shown in Sup. Fig. 2 (Column 2), even though the random pattern itself moves to the right.

Fluted Square Wave A square wave moving to the right, a quarter cycle every frame, elicits the correct response (right) as shown in Sup. Fig. 3 (Column 1). When the fundamental frequency of the square wave is removed, it results in a moving "fluted square wave", which elicits a significant amount of motion energy in the opposite (left) direction as shown in Sup. Fig. 3 (Column 2).

These results show that our implementation reproduces outputs characteristic of the spatiotemporal energy model (Adelson & Bergen, 1985). We also provide full input sequences and results for above validation along with the code: `https://github.com/aravindbattaje/motion_perception_tool/tree/main/validation`.

References

Adelson, E. H., & Bergen, J. R. (1985, Feb). Spatiotemporal energy models for the perception of motion. *J. Opt. Soc. Am. A*, *2*(2), 284–299. doi: 10.1364/JOSAA.2.000284

Adelson, E. H., & Movshon, J. A. (1986, January). The perception of coherent motion in two-dimensional patterns: 1986 ACM SIGGRAPH/SIGART Interdisciplinary Workshop on Motion: Representation and Perception. *Proceeding of the ACM SIGGRAPH/SIGART Interdisciplinary Workshop on Motion*, 93–98. (Publisher: Association for Computing Machinery, Inc)

Anstis, S. M. (1970, December). Phi movement as a subtraction process. *Vision Research*, *10*(12), 1411–IN5. Retrieved 2022-12-06, from https://www.sciencedirect.com/science/article/pii/0042698970900921 doi: 10.1016/0042-6989(70)90092-1

Anstis, S. M., & Rogers, B. J. (1986, October). Illusory Continuous Motion from Oscillating Positive-Negative Patterns: Implications for Motion Perception. *Perception*, *15*(5), 627–640. Retrieved 2022-12-06, from https://doi.org/10.1068/p150627 (Publisher: SAGE Publications Ltd STM) doi: 10.1068/p150627

Challinor, K. L., & Mather, G. (2010, June). A motion-energy model predicts the direction discrimination and MAE duration of two-stroke apparent motion at high and low retinal illuminance. *Vision Research*, *50*(12), 1109–1116. Retrieved 2022-12-01, from https://linkinghub.elsevier.com/retrieve/pii/S0042698910001586 doi: 10.1016/j.visres.2010.04.002

Finlay, D., Dodwell, P., & Caelli, T. (1984, June). The Waggon-Wheel Effect. *Perception*, *13*(3), 237–237. Retrieved 2022-12-06, from https://doi.org/10.1068/p130237 (Publisher: SAGE Publications Ltd STM) doi: 10.1068/p130237

Mather, G., & Challinor, K. L. (2009, January). Psychophysical properties of two-stroke apparent motion. *Journal of Vision*, *9*(1), 28–28. Retrieved 2022-12-01, from http://jov.arvojournals.org/Article.aspx?doi=10.1167/9.1.28 doi: 10.1167/9.1.28

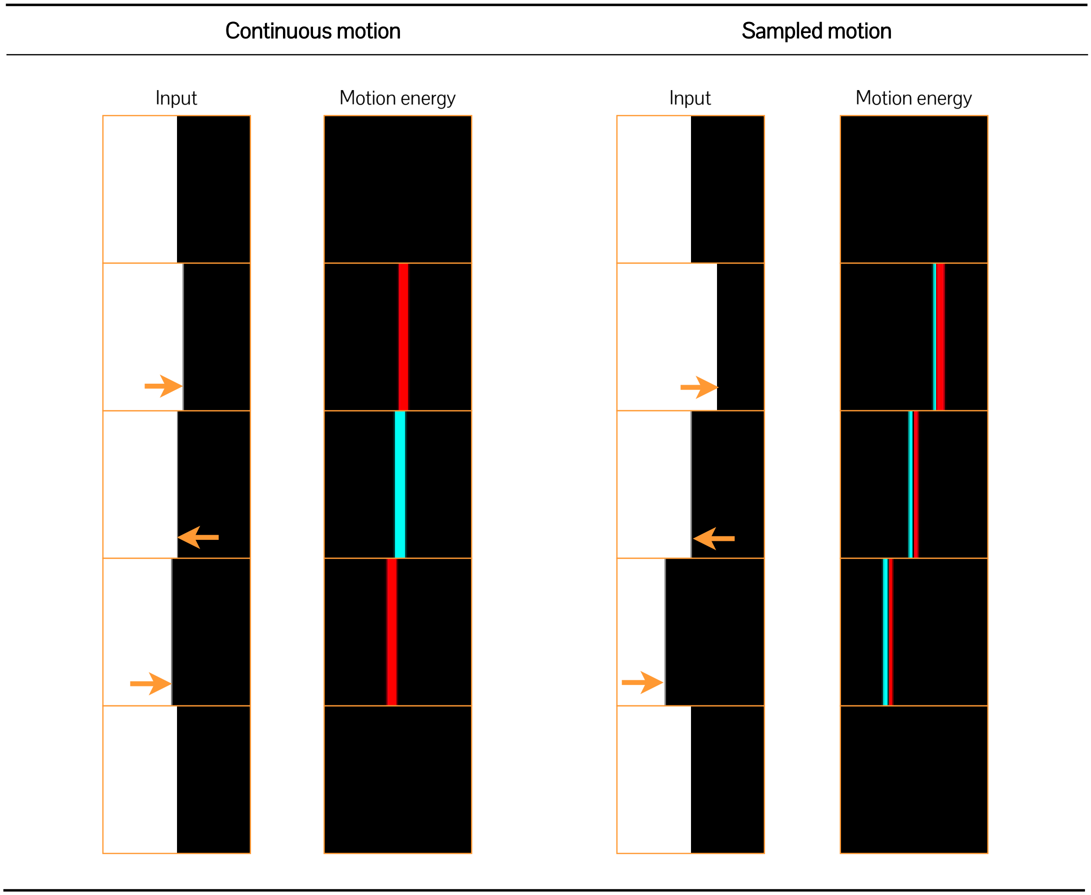

Supplementary Figure 1. Motion energy output is qualitatively similar to (Adelson & Bergen, 1985) for continuous and sampled motion stimuli. For both types of motion, the border separating white and black regions of the stimulus is first static, then moves in a sinusoidal fashion (in left-right direction), and comes to a stop. Each row shows the input and model response (center-cropped to 2.4x magnification for visual clarity) for some representative times during such a sequence. The arrows in the input column indicate direction of movement of the border, and the colors in motion energy column indicate direction—red for right, and cyan for left.
In continuous motion where the border moves sufficiently slowly to seem continuous, the motion energy shows only-rightward or only-leftward motion when the border moves. However, for sampled motion where the border moves relatively faster, it elicits some motion in the opposite direction, similar to the wagon-wheel effect (Finlay, Dodwell, & Caelli, 1984).

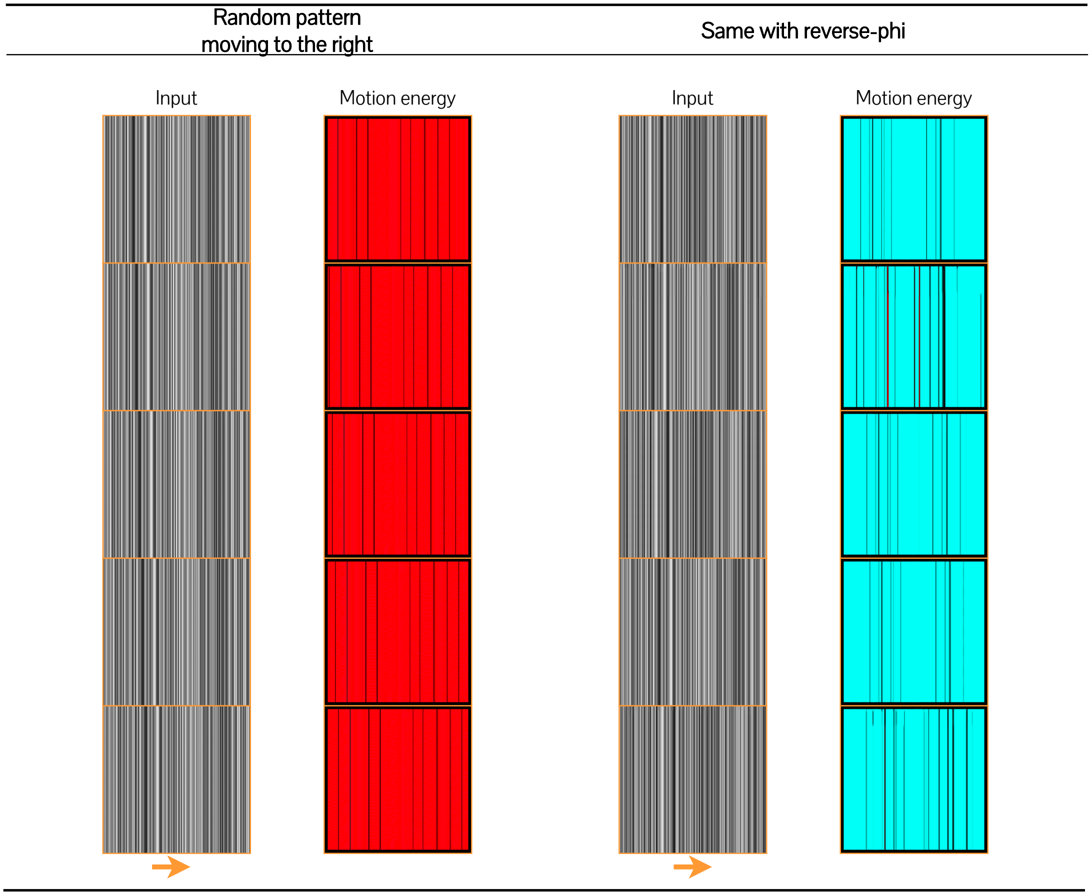

Supplementary Figure 2. Motion energy output is qualitatively similar to (Adelson & Bergen, 1985) for stimuli without and with reverse-phi (Anstis, 1970; Anstis & Rogers, 1986). For both conditions, a random pattern (in X) is moved slowly to the right. Each row shows the input and motion energy at increasing times. For visual clarity, every 5th frame from the input sequence is shown per row. For the moving random pattern with reverse-phi, the polarity of every other frame (and hence also every 5th frame) is reversed. The arrows at the bottom of input column indicate actual direction of motion for both patterns, and the colors in motion energy column indicate direction—red for right, and cyan for left.
In the case of random pattern moving to the right, motion energy shows rightward movement in the entire visual scene, and when contrast inversion is applied to the same rightward moving pattern, motion energy switches to the left direction, replicating reverse-phi.

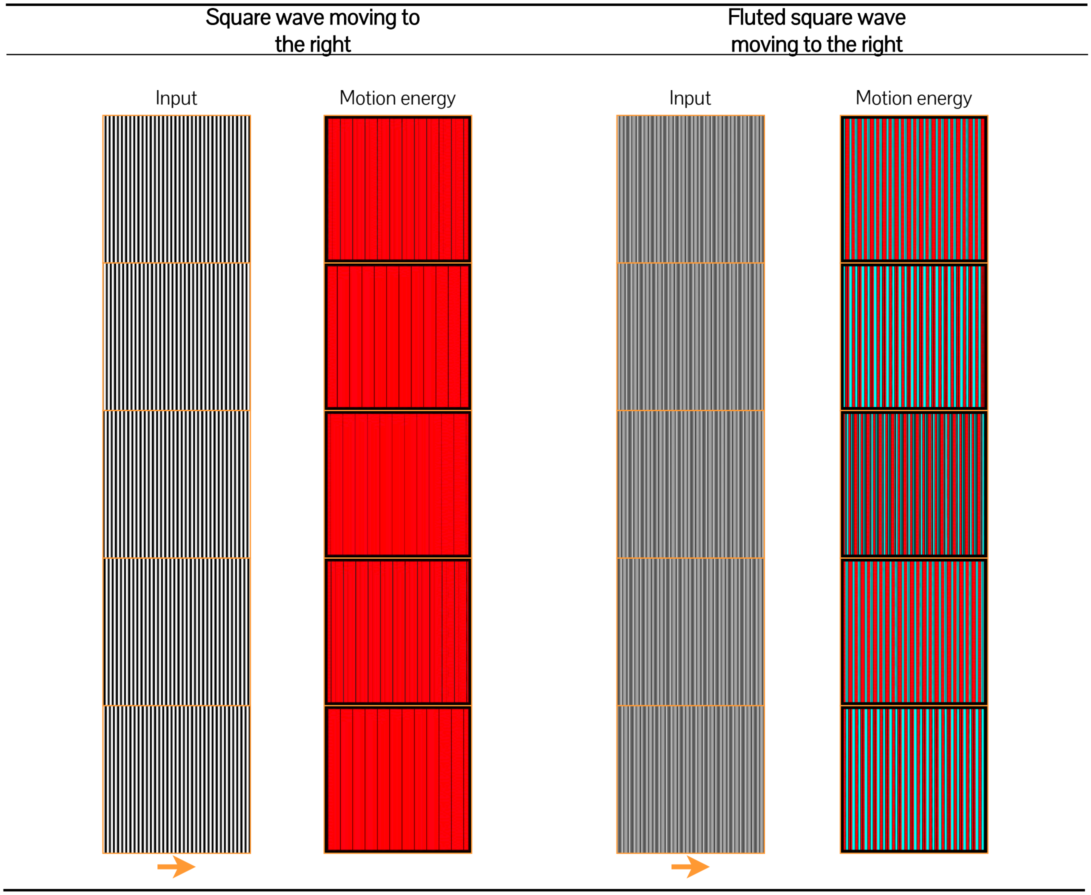

Supplementary Figure 3. Motion energy output is qualitatively similar to (Adelson & Bergen, 1985) for square wave and fluted square wave. Each row in the figure correspond to consecutive frames in the input sequence. Both square wave and fluted square wave (in X) move to the right a quarter cycle every frame. The fluted square wave is constructed from a square wave by removing its first fundamental frequency. The arrows at the bottom of input column indicate actual direction of motion for both patterns, and the colors in motion energy column indicate direction—red for right, and cyan for left.
As expected for a square wave moving to the right, motion energy shows rightward movement. However for fluted square wave, there is also significant energy in the opposite direction.
